# Epidural anesthesia and cancer outcomes in bladder cancer patients: is it the technique or the medication? A matched-cohort analysis from a tertiary referral center

**DOI:** 10.1186/s12871-018-0622-5

**Published:** 2018-11-03

**Authors:** Juan Chipollini, Brandon Alford, David C. Boulware, Patrice Forget, Scott M. Gilbert, Jorge L. Lockhart, Julio M. Pow-Sang, Wade J. Sexton, Philippe E. Spiess, Michael A. Poch, Sephalie Y. Patel

**Affiliations:** 1 Department of Genitourinary Oncology, Moffitt Cancer Center, 12902 Magnolia Drive, Tampa, FL 33612 USA; 2 Morsani College of Medicine, University of South Florida, 12901 Bruce B. Downs Blvd, Tampa, FL 33612 USA; 3 Department of Biostatistics and Bioinformatics, Moffitt Cancer Center, 12902 Magnolia Drive, Tampa, FL 33612 USA; 4 Department of Anesthesiology, Vrije Universiteit Brussel (VUB), Universitair Ziekenhuis, Laarbeeklaan 101, 1090 Brussels, Belgium; 5 Department of Anesthesiology, Moffitt Cancer Center, 12902 Magnolia Drive, Tampa, FL 33612 USA

**Keywords:** Cystectomy, Epidural anesthesia, Perioperative period, Retrospective review

## Abstract

**Background:**

The perioperative period can be a critical period with long-term implications on cancer-related outcomes. In this study, we evaluate the influence of regional anesthesia on cancer-specific outcomes in a radical cystectomy (RC) cohort of patients.

**Methods:**

We performed a retrospective analysis of patients with clinically-nonmetastatic urothelial carcinoma of the bladder who underwent RC at our institution from 2008 to 2012. Patients were retrospectively registered and stratified based on two anesthetic techniques: perioperative epidural analgesia with general anesthesia (epidural) versus general anesthesia alone (GA). Epidural patients received a sufentanil-based regimen (median intraoperative sufentanil dose 50 mcg (45,85). Propensity-score was used to make 1:1 case-control matching. Cumulative risk of recurrence with competing risks was calculated based on anesthetic technique. Kaplan-Meier curves were used to compare recurrence-free (RFS) and cancer-specific survival (CSS). Univariable and multivariable analyses were performed with Cox proportional hazard regression models for RFS and CSS.

**Results:**

Only patients with complete data on anesthetic technique were included. Out of 439 patients, 215-pair samples with complete follow-up were included in the analysis. Median follow-up was 41.4 months (range: 0.20–101). Patients with epidurals received higher median total intravenous morphine equivalents (ivMEQ) versus those in the GA group (75 (11–235) vs. 50 ivMEQ (7–277), *p* < 0.0001). Cumulative risk of recurrence at two years was 25.2% (19.6, 31.2) for epidural patients vs. 20.0% (15.0, 25.7) for GA patients (Gray test *p* = 0.0508). Epidural analgesic technique was a significant predictor of worse RFS (adjusted HR = 1.67, 1.14–2.45; *p* = 0.009) and CSS (HR = 1.53, 1.04–2.25; *p* = 0.030) on multivariable analyses.

**Conclusions:**

Epidural anesthesia using sufentanil was associated with worse recurrence and disease-free survival in bladder cancer patients treated with surgery. This may be due use of epidural sufentanil or due to the increased total morphine equivalents patient received as a consequence of this drug.

## Background

The perioperative period is a critical period which may impact long-term cancer outcomes. A complex state of inflammation, immunosuppression, angiogenesis, and high adrenergic state has been shown to potentially facilitate growth of residual disease and promote seeding of circulating tumor cells [1–3]. Thus, tumor surveillance and eradication are strongly dependent on an adequate immune response to these potentially influencing tumor biology [4–6].

Radical cystectomy (RC) with pelvic lymph node dissection is the mainstay treatment for muscle invasive bladder cancer. A greater survival advantage has been shown at 2 years or more following RC as opposed to bladder-sparing modalities [7]. However, the surgical stress response might initiate a cascade of cellular, hormonal, and neuronal perturbations that leave patients susceptible to the pro-metastatic effects of tumor manipulation [4–6]. In addition, the choice of anesthetic technique may influence cancer outcomes in two ways. First, the immosuppressive effects of opioids, has been proposed to possibly influence immunogenicity during surgery in several solid organ malignancies thereby influencing cancer recurrence [8, 9]. Secondly, regional anesthesia such as epidural anesthesia may blunt the pro metastatic effect of surgical stress [10, 11].

If one can attenuate these perioperative pro-metastatic processes, the immediate postoperative period could become a window of opportunity to improve long-term oncologic control for surgical oncology patients. Given the paucity of studies in the urological and anesthetic literature, we aim to evaluate cancer outcomes in a matched cohort of patients undergoing RC at our institution. Our hypothesis was that tumor recurrence may be associated with the use of epidural opioids.

## Methods

### Analgesia management and study population

The study was approved by institutional review board. We retrospectively reviewed the medical records of all patients who underwent RC from January 2008 and December 2012 for clinically non-metastatic urothelial carcinoma of the bladder with curative intent. Patients were stratified based on anesthetic technique which included a standardized protocol of general endotracheal anesthesia with epidural (epidural group) or general endotracheal anesthesia alone (GA group). Epidural catheters were placed at the discretion of the anesthesiologist and surgeon. Catheters were placed between T10-T12 levels and dosed primarily with sufentanil during the intra and postoperative period (median intraoperative dose = 50mcg, range 45–85). Patients without epidural catheters utilized patient-controlled analgesia with either intravenous morphine or hydromorphone.

**Fig. 1:**
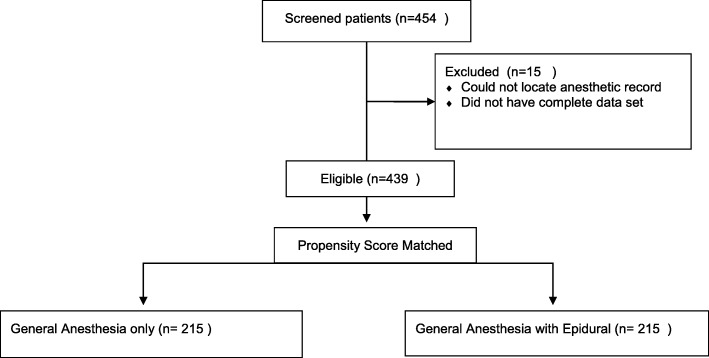
Patient Flow Diagram

Propensity score matching based on age, American Society of Anesthesiologists (ASA) score, and pathologic N and T stage was performed for those patients with complete analgesic data and surveillance follow-up. In total, 215 matched subjects from each cohort was produced and included in the final analysis (Fig. 1). A data driven approach was not used in selecting characteristics to adjust for, rather the authors chose factors which might cause an imbalance in comparing the two cohorts in subsequent analysis. We agree that a data-driven approach could have been used, but there were no significant differences when comparing cohorts. Additionally, propensity matching was performed using anesthesia type as the outcome, not survival. Consequently, the matching does not preclude effects of measured and unmeasured baseline characteristics. For this reason we also adjusted the multivariable models for these effects.

### Primary outcomes

Primary outcomes measured were recurrence-free (RFS) and cancer-specific survival (CSS). Survival was assigned from date of surgery until date of recurrence (RFS) or death from disease (CSS). Follow-up data were available through our institution’s tumor registry, which tracks patients’ death certificates and follow-up at other medical facilities. Reasons for censoring were lost of follow up and death from any other known cause with documented absence of detectable disease after regular follow up.

To investigate whether or not total perioperative use of systemic opioids was different, we compared total intravenous morphine equivalents (ivMEQ) given in the intraoperative and immediate postoperative period. The ivMEQ were calculated on the basis of 1 mg morphine = 1mcg sufentanil, 0.2 mg hydromorphone, and 10 mcg fentanyl [12, 13].

### Statistical analysis

Frequencies, means, medians, and ranges were used in the descriptive tables to characterize the data both in total and by anesthesia type. For a subgroup of the variables, nonparametric tests (Wilcoxon rank sum test or Kruskal Wallis test for continuous and Fisher’s Exact test for categorical variables, respectively) were performed comparing anesthesia cohorts. Cumulative risk of recurrence with competing risks analysis was calculated based on anesthetic technique and compared with the Gray test. Death was the competing event. The Kaplan-Meier method was used to estimate RFS and CSS. Cox Proportional Hazards regression was used in both univariable and multivariable models for each endpoint. For CSS, backwards selection process was performed. For RFS, competing risk analysis was carried out. All analyses were performed in either SAS (v 9.4) and R software. This study is written in accordance to the Strengthening Reporting in Observational and Epidemiological studies (STROBE) guidelines (www.strobe-statement.org).

## Results

### Study cohort characteristics

Demographic and tumor characteristics of the entire cohort are provided in Table 1. Overall, 77.2% of patients were male. Median age was 69 years and median clinical follow-up was 41.4 months (range: 0.20–101). Approximately 29% and 20% received neoadjuvant and adjuvant systemic treatments, respectively. The most common urinary diversion was the ileal conduit (65.1%). Approximately 44 and 26% had pT3/T4 and pN+ disease, respectively. The positive radial and ureteral or urethral margin rates were 12.3 and 6.5%, respectively.

**Table 1:** Characteristics of the sample (*n* = 430 patients)

Variable	Level	*N* = 430	%
Gender	Male	332	77.2
	Female	98	22.8
Age (years)	Median	69	–
	Std Dev	10.04	–
BMI (kg/m^2^)	Median	27.85	–
	Std Dev	5.27	–
Race	White	404	94.0
	Black	14	3.3
	Asian	1	0.2
	Native Hawaiian/Pacific Islander	1	0.2
	American Indian/Alaskan	1	0.2
	Unknown/Other	9	2.1
ASA score	1	3	0.7
	2	194	45.1
	3	224	52.1
	4	9	2.1
Anesthesia groups	General only	215	50.0
	General + Epidural	215	50.0
Neoadjuvant chemotherapy	No	305	71.4
	Yes	122	28.6
	Unknown	3	–
Diversion Type	Ileal conduit	280	65.1
	Continent catherizable pouch	15	3.5
	Neobladder	67	15.6
	Cutaneous ureterostomy	63	14.6
pT stage	T0	59	13.7
	T1	44	10.2
	T2	82	19.1
	T3	125	29.1
	T4	62	14.4
	Ta/Tis	58	13.5
pN stage	Nx	19	4.4
	N1	35	8.1
	N2	60	14.0
	N3	17	4.0
	N0	299	69.5
Radial margin	Negative	377	87.7
	Positive	53	12.3
Ureteral or urethral margin	Negative	402	93.5
	Positive	28	6.5
Grade	High	350	96.7
	Low	5	1.4
	Mixed	2	0.6
	Missing	73	–
Adjuvant chemotherapy	No	340	79.8
	Yes	86	20.2
	Missing	4	–
Location of Recurrence	Pelvic	22	16.5
	Upper tract	11	8.3
	Distant	97	72.9
	Other	3	2.3
	None	297	–

The non-epidural cohort had more male patients (81.9 vs 72.6%) (*p* = 0.021), as well as more patients with T3/4 tumor stage (47.4 vs 39.5%) (*p* = 0.096). No significant differences were found for hospital length of stay, race, ethnicity, body mass index (BMI), median follow-up, node stage or chemotherapy status between the two groups (Table 2).

**Table 2:** Characteristics of the cohorts with tests (*n* = 430)

Covariate	Level	GA (Non-Epidural) *N* = 215	Epidural *N* = 215	*P*-value
Gender	Male	176 (81.86)	156 (72.56)	**0.021**
	Female	39 (18.14)	58 (27.44)	
Age (years)	Median	70	69	0.384
Follow-up (months)	Median	40.7	42.9	0.260
Race	Unknown/Other	7 (3.26)	2 (0.93)	0.181
	White	197 (91.63)	207 (96.28)	
	Black	8 (3.72)	6 (2.79)	
	Asian	1 (0.47)	0 (0)	
	Hawaiian/P. Islander	1 (0.47)	0 (0)	
	Indian/Alaskan	1 (0.47)	0 (0)	
Ethnicity	Unknown/Other	138 (64.19)	140 (66.12)	0.977
	Not Hispanic/Latino	73 (33.95)	71 (33.02)	
	Hispanic/Latino	4 (1.86)	4 (1.86)	
ASA	1	1 (0.47)	2 (0.93)	0.147
	2	86 (40)	108 (50.23)	
	3	123 (57.21)	101 (46.98)	
	4	5 (2.33)	4 (1.86)	
Length of stay (days)	Median	8	7	0.352
BMI (kg/m^2^)	Median	28.46	27.75	0.191
pT stage	T0	31 (14.42)	28 (13.02)	0.096
	T1	20 (9.3)	24 (11.16)	
	T2	30 (13.95)	52 (24.19)	
	T3	71 (33.02)	54 (25.12)	
	T4	31 (14.42)	31 (14.42)	
	Ta/Tis	32 (14.88)	26 (12.09)	
pN stage	Nx	10 (4.65)	9 (4.19)	0.808
	N1	19 (8.84)	16 (7.44)	
	N2	33 (15.35)	27 (12.56)	
	N3	7 (3.26)	10 (4.65)	
	N0	146 (67.91)	153 (71.16)	
Neoadjuvant chemotherapy	No	155 (72.77)	150 (70.09)	0.540
	Yes	58 (27.23)	64 (29.91)	

**Fig. 2:**
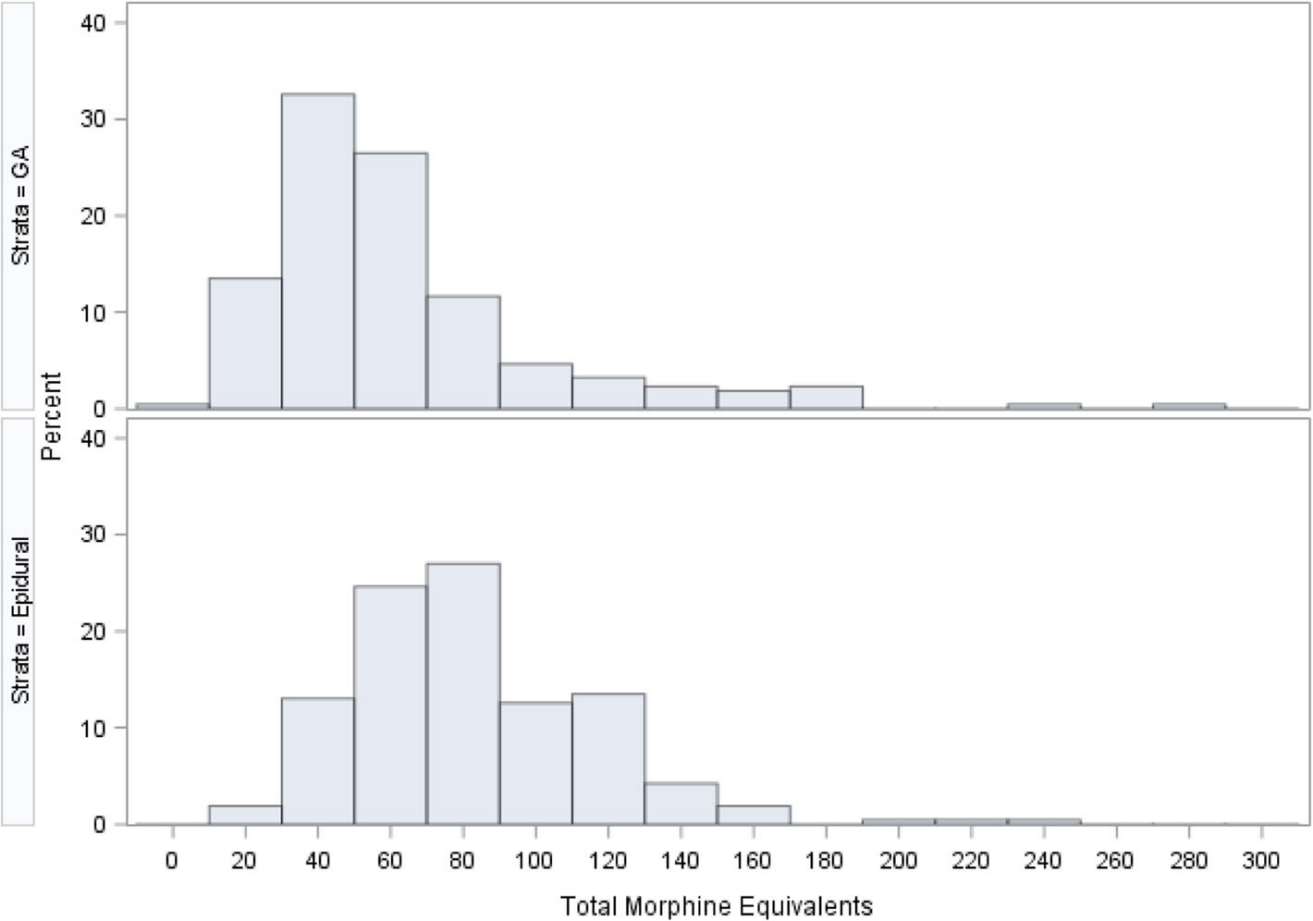
Histogram of Total Morphine Equivalents by Anesthetic technique

Patients in the epidural group had higher median total morphine equivalents versus those in the GA group (75 (11–235) vs 50 ivMEQ (7–277), *p* < 0.0001) (Fig. 2). None of the patients received intraoperative non-steroidal anti-inflammatory drugs (NSAID). Postoperatively, the NSAID use was not available.

### Recurrence rates

**Fig. 3:**
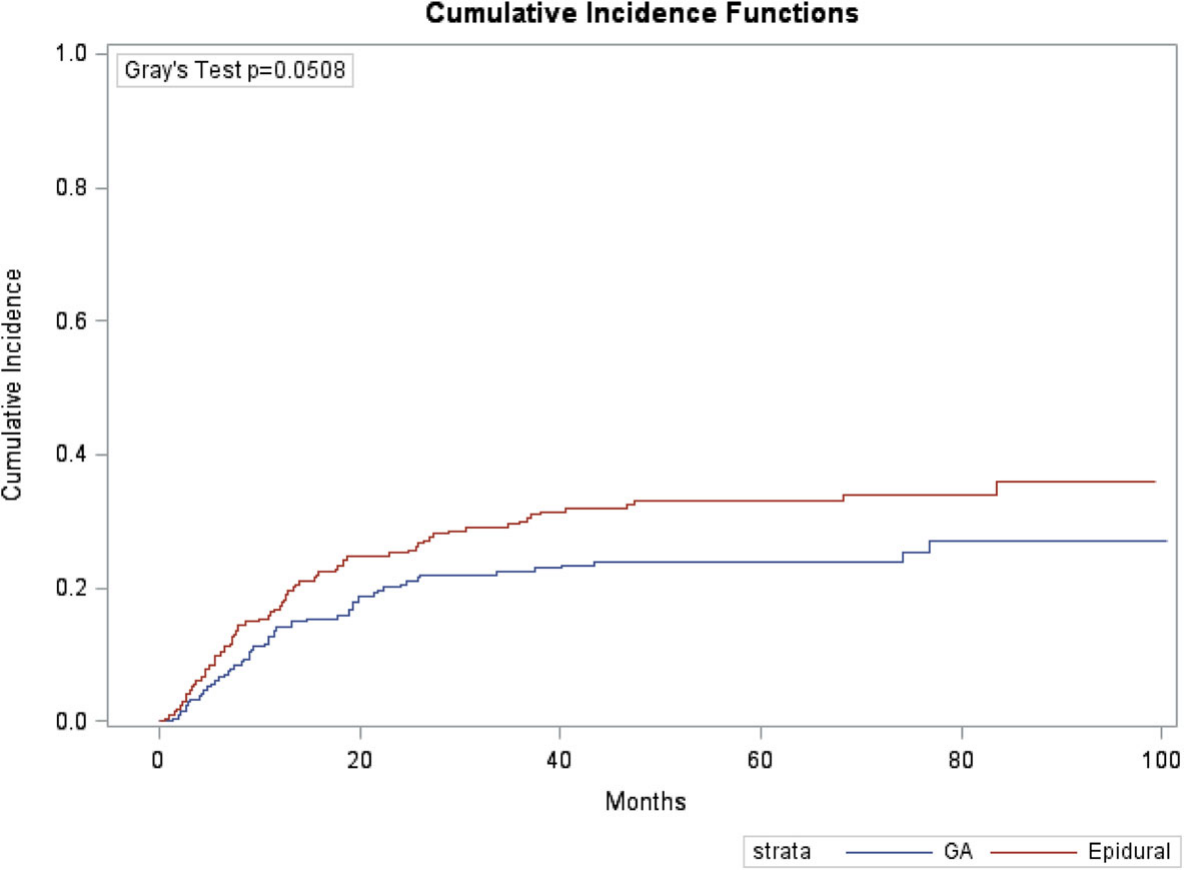
Cumulative risk of recurrence between the two groups

Of the 430 patients, 133 (30.9%) developed a recurrence with the most common type being distant metastatic disease (72.9%). A cumulative risk for recurrence plot is presented in Fig. 3. The median time to recurrence was 11 and 11.8 months for epidural and GA, respectively. Majority of recurrences occurred within 2 years after RC with rates of 20.0% (95%CI: 15.0–25.7) and 25.2% (19.6–31.2) for GA vs. epidural patients, respectively (Gray test *p* = 0.051).

### Survival analysis

**Fig. 4:**
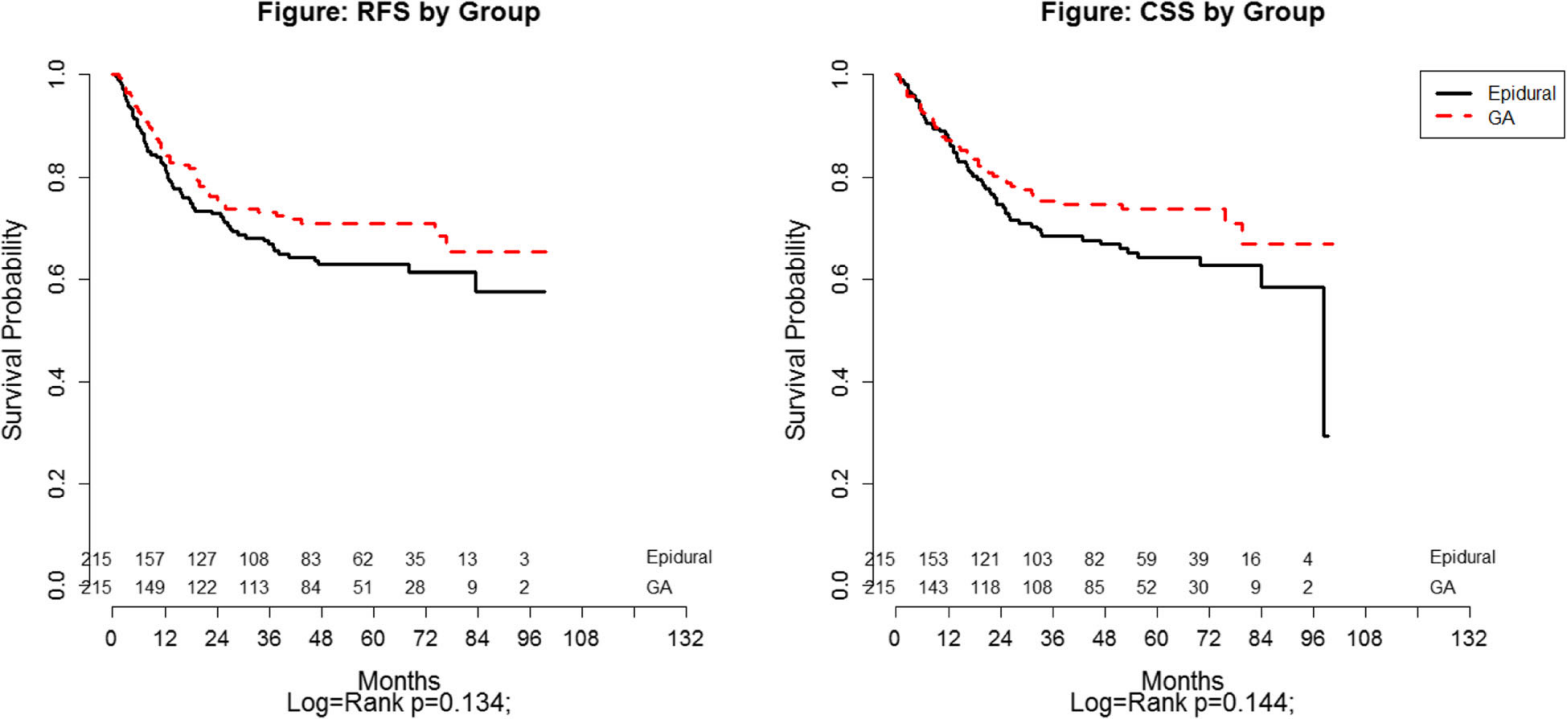
Recurrence-free (RFS) and Cancer-specific (CSS) survival by Anesthetic technique

Figure 4 shows survival curves based on anesthesia type. Five-year RFS and CSS for the whole cohort were 67.2% (62.0–71.8) and 68.7% (63.3–73.5), respectively. Epidural patients had a 5-year RFS of 62.9% (55.4–69.4) vs. 70.9% (63.5–77.2) for GA patients (log-rank *p* = 0.134). Five- year CSS were 64.2% (95% CI 56.2–71.1) and 73.7% (95% CI 66.0–79.8) (log-rank: *p* = 0.144) for epidural vs. GA patients, respectively.

### Predictors for RFS and CSS

Tables 3 and 4 show the results of Cox proportional hazards modeling for RFS and CSS, respectively. When adjusting for relevant covariates, patients who received epidurals were at increased risk of recurrence (adjusted HR = 1.67, 1.14–2.45; *p* = 0.009) and death of disease (HR = 1.53, 1.04–2.25; *p* = 0.030). Other predictors of recurrence were age (adjusted HR = 0.98, *p* = 0.045), pT stage (adjusted HR = 2.22, *p* < 0.001), and neoadjuvant chemotherapy (adjusted HR = 1.66, *p* = 0.009) while pN stage (HR = 2.48, *p* < 0.001), pT stage (HR = 3.63, *p* <  0.001), and margin status (HR = 2.00, *p* = 0.012) were associated with worse cancer survival.

**Table 3:** Cox proportional hazards regression for recurrence-free survival (*n* = 430) with competing risks

Covariate	Level	Univariable Hazard Ratio (95% CI)	*P*-value	Multivariable Hazard Ratio (95% CI)	*P*-value
Age		0.98 (0.96–0.99)	**0.020**	0.98 (0.98–1.00)	**0.045**
Gender	Male	1.36 (0.86–2.14)	0.190	1.62 (0.99–2.66)	0.057
	Female	Ref			
ASA	≥ 3	0.81 (0.57–1.15)	0.232	0.91 (0.62–1.33)	0.620
	≤ 2	Ref			
Anesthesia	GA + Epidural	1.43 (1.00–2.04)	**0.048**	1.67 (1.14–2.45)	**0.009**
	GA	Ref			
pN stage	N+	1.76 (1.21–2.55)	**0.003**	1.24 (0.82–1.88)	0.308
	N0/Nx	Ref			
pT stage	≥ T3	2.47 (1.74–3.52)	**< 0.001**	2.22 (1.43–3.46)	**0.0004**
	≤ T2	Ref			
Margin (radial/ureteral/urethral)	Positive	1.42 (0.95–2.12)	0.091	1.23 (0.78–1.94)	0.370
	Negative	Ref			
Neoadjuvant chemotherapy	Yes	1.69 (1.18–2.43)	**0.005**	1.66 (1.14–2.43)	**0.009**
	No	Ref			
Adjuvant chemotherapy	Yes	2.17 (1.49–3.16)	**< 0.001**	1.34 (0.85–2.11)	0.206
	No	Ref			

Bold value indicates statistical significance

**Table 4:** Cox proportional hazards regression for cancer-specific survival (*n* = 430)

Covariate	Level	Univariable Hazard Ratio (95% CI)	*P*-value	^a^Multivariable Hazard Ratio (95% CI)	*P*-value
Age		0.99 (0.97–1.01)	0.448		
Gender	Male	1.14 (0.70–1.85)	0.600		
	Female	Ref			
ASA	≥ 3	1.24 (0.85–1.82)	0.256		
	≤ 2	Ref			
Anesthesia	GA + Epidural	1.33 (0.91–1.95)	0.146	1.53 (1.04–2.25)	**0.030**
	GA	Ref			
pN stage	N+	3.70 (2.52–5.43)	**< 0.001**	2.48 (1.64–3.74)	**< 0.001**
	N0/Nx	Ref			
pT stage	≥ T3	4.98 (3.31–7.51)	**< 0.001**	3.63 (2.32–5.68)	**< 0.001**
	≤ T2	Ref			
Margin (radial/ureteral/urethral)	Positive	2.38 (1.56–3.62)	**< 0.001**	2.00 (1.29–3.09)	**0.012**
	Negative	Ref			
Neoadjuvant chemotherapy	Yes	1.34 (0.90–1.99)	0.151		
	No	Ref			
Adjuvant chemotherapy	Yes	2.03 (1.35–3.04)	**0.0006**		
	No	Ref			

^a^Results are from a backwards selection process (significance level at 0.05)

Bold value indicates statistical significance

## Discussion

Patients who received epidural analgesia sustained a decreased time to disease recurrence. Interestingly the log rank *p*-values (Fig. 4) did not show a statistical difference for RFS and CSS between groups, however the univariate Cox proportional hazard regression showed a statistical difference in RFS between the epidural and GA group. This is mostly likely due to the use of competing risk methods that was used in the RFS analysis. Since epidural sufentanil is such a potent opioid translating to a large ivMEQ, we believe the immunosuppressive effects of increased systemic opioids led to the poor recurrence and survival outcomes seen in our epidural cohort of patients [14].

The perioperative period is a fragile state for patients undergoing cancer surgery and is characterized by various pro-metastatic processes such as release of tumor cells, increased levels of pro-angiogenic growth factors, and numerous hormonal and autonomic alterations caused by the surgery-induced stress response [15]. Thus, it is the synchronization of these detrimental processes that may render a patient susceptible to poor oncologic outcomes. Given that anesthesia and analgesia play an integral role in perioperative recovery and pain control, our study is significant in describing an association between anesthetic technique and cancer outcomes for bladder cancer patients.

Surgery induces a stress response that can affect the host’s cytotoxic T-cell and natural killer (NK) cellular function, increase pro-angiogenic factors that promote tumor growth, and reduce circulating concentrations of tumor-related anti-angiogenic factors [16–19] In addition, systemic opioids used during hospital course can affect both humoral and cellular immunity by inhibiting antibody production, NK cell activity, cytokine expression, and phagocytic activity [14, 20–22].

One previous study of over 1000 prostatectomy patients reported intravenous sufentanil administration associated with increased risk of biochemical recurrence [8]. Interestingly, 97% of patients received sufentanil (epidural and intravenous) introducing a risk of type 1 error. Patients receiving intravenous sufentanil had a significant reduced survival rate (HR 7.8). The authors did not compare the dosage of sufentanil given through the epidural versus intravenously so we are unable to tell if the dosage was equal in both groups. If the intravenous sufentanil group received a higher ivMEQ, this would confirm previous observations that synthetic lipophilic opioids can negatively impact cancer-related outcomes. Given that our epidural cohort did not receive the opioid-sparing effect that is normally seen with hydrophilic opioid-based regional analgesia, our study adds support for decreased use of systemic opioids for patients recovering from cancer surgeries. Due to the inhibitory effects of opioids on not only NK cell function but also immunological pathways involving monocytes, secretion of immunoglobulins, cytokines and cell proliferation [8, 23], the increased systemic absorption caused by lipophilic, potent analgesics such as sufentanil may be detrimental for our cystectomy patients.

The issue of opioid use in the perioperative period has been increasingly recognized in the literature. The role of the mμ opioid receptor (MOR), which has been shown expressed in cancer cells such lung adenocarcinoma and breast cancer, has been postulated as a mechanism of cancer progression due its interactions with angiogenesis, epithelial-mesenchymal transition, and the mammalian target of rapamycin signaling pathways [24–26]. These findings have been substantiated by retrospective data suggesting worse outcomes in patients with cancer who are exposed to systemic opioids and high levels of MOR expression [24, 27, 28]. Although our study adds more evidence on the detrimental effect of synthetic opioids, our findings require further translational studies which may help further characterize the pathophysiological basis of MOR inhibition and identify potential targets for future therapeutic strategies.

Several important limitations are inherent in this study including a limited sample size and the retrospective, observational design. Although we used propensity score matching to reduce selection bias, the effects of unmeasured confounding variables cannot be excluded. Our results relate to only sufentanil epidural anesthesia and do not translate to other neuraxial anesthetic techniques. Additionally, any association between anesthetic techniques and oncologic outcomes among patients with bladder cancer will need to be better characterized from a pathophysiological standpoint for our results to have not only epidemiological but therapeutic implications.

## Conclusions

Worse oncologic outcomes may be associated with sufentanil based epidural anesthesia during radical cystectomy. This may have been due to the larger amount of opioids used with epidural sufentanil. Although limited by its retrospective design, our study suggests that further investigation in the clinical setting evaluating the effects of opioid-based epidural analgesia and bladder cancer progression is warranted.

## Abbreviations

ASA: American Society of Anesthesiologists
BMI: Body Mass Index
CSS: Cancer Specific Survival
GA: General Anesthesia
HR: Hazard Ratio
ivMEQ: Intravenous morphine equivalents
MOR: Mu opioid receptor
NK: Natural Killer
NSAID: Non Steroidal Anti Inflammatory Drug
RC: Radical Cystectomy
RFS: Recurrence Free Survival
STROBE: Strengthening Reporting in Observational and Epidemiological Studies
